# Supplementary material for: Physical confinement and phagocytic uptake induce persistent cell migration
Source: Biol Open. 2025 Sep 17;14(9):bio062021. doi: 10.1242/bio.062021 (PMC12486203; doi:10.1242/bio.062021)
Supplement: Supplementary information [file biolopen-14-062021-s1.pdf]

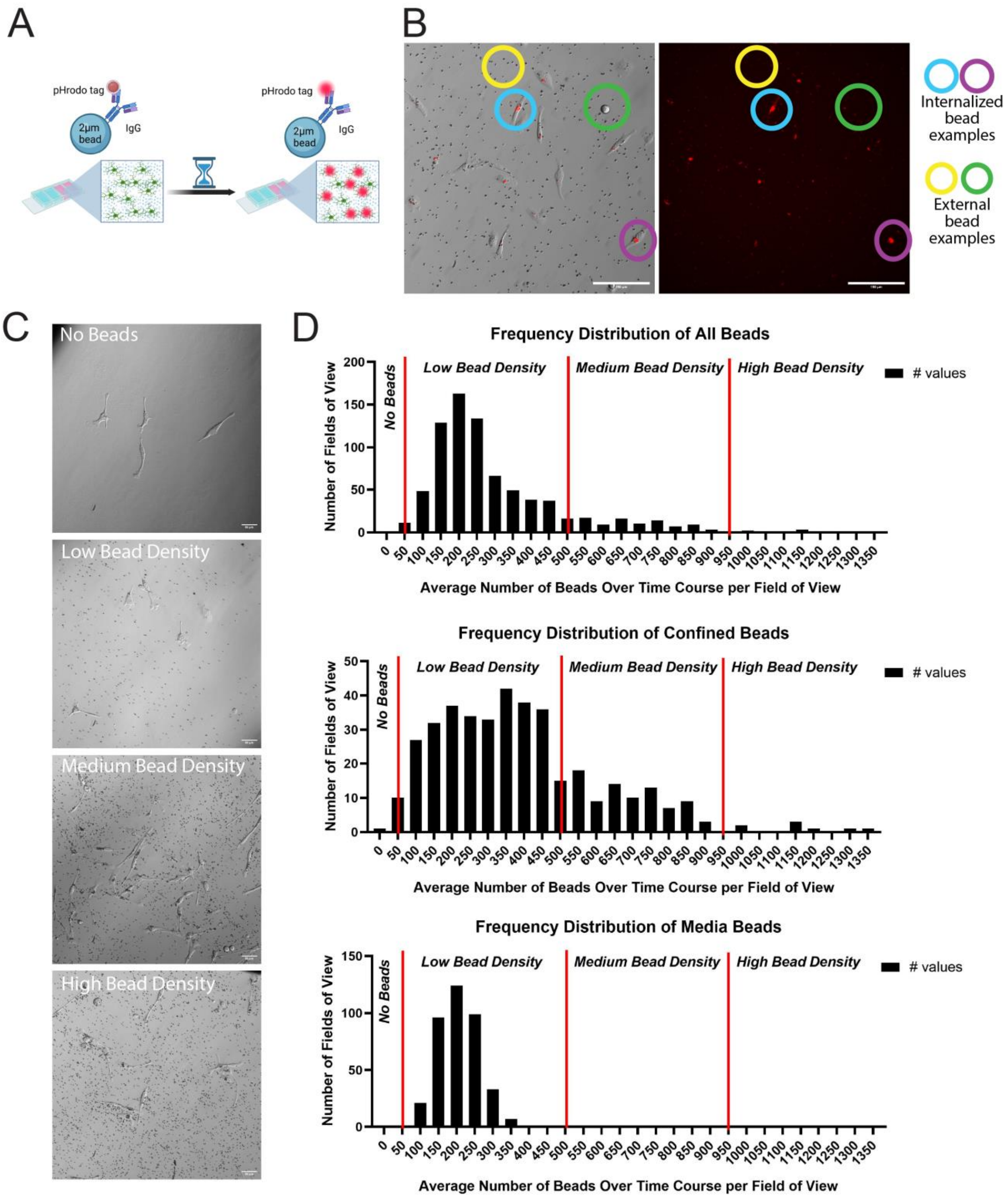

Fig. S1. Strategy for controlling for bead density. A) Schematic depicting phagocytic bead labelling and the pHrodo tag fluorescing inside cells but not externally. B) Duplication of Figure 2A. Examples of either internalized (blue and purple circles) or external (yellow and green

Biology Open • Supplementary information

circles) pHrodo-red staining outlined in both composite and pHrodo only images. Scale bar represents 100µm. C) Example phase contrast images displaying different bead densities. No beads (top) through high bead density (bottom). Scale bar represents 50µm. D) Histograms detailing the breakdown of average bead densities per field of view across all experimental runs. Low bead density was classified as any field of view with a bead average between 50 and 500 beads; medium bead density was 500 to 950 beads; high bead density was any field of view average above 950 beads. Low bead densities in confined images most closely matched the density of typical media images. Created in BioRender by Paulson, S. (2025) https://BioRender.com/r1d8ldk

Biology Open • Supplementary information

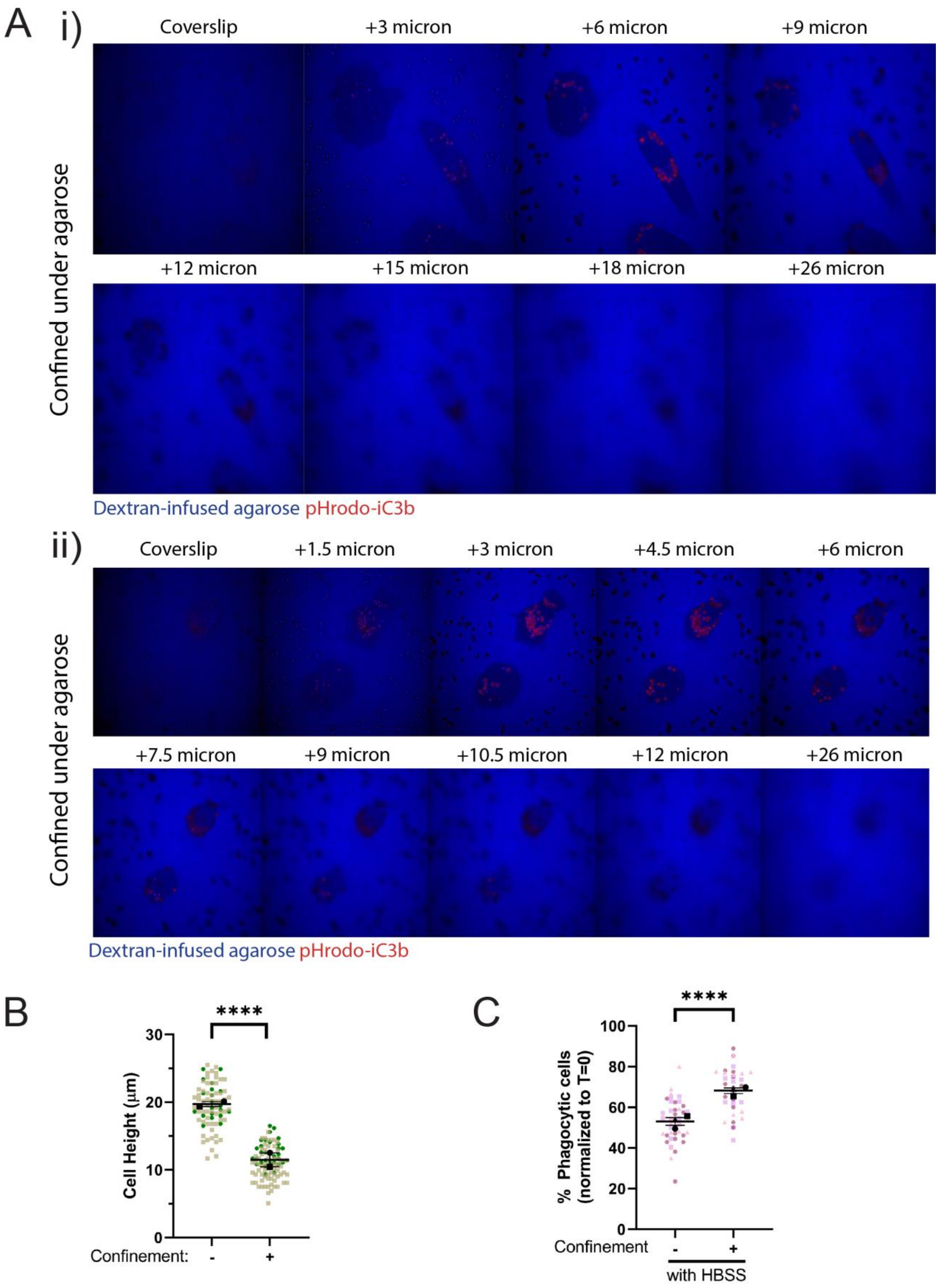

Biology Open • Supplementary information

Fig. S2. Confirming confinement after bead introduction, and ruling out influence of HBSS. A) Example image series of cell height under dextran-infused agarose confinement from two different fields of view (i and ii). Cell outlines are visible through an absence of cascade blue labelled dextran (Blue), and the presence of pHrodo-red beads (Red). B) Cell heights calculated through Z stack imaging in media or under-confinement. Each z-step is 0.3 µm, and this value was multiplied by the number of frames containing cells across the z stack. C) Percent phagocytic cells at 2 hours in either media containing HBSS or normal agarose (containing HBSS). For all graphs, black points demonstrate experiment means, colored points demonstrate individual cell values for each run. N = 2 experiments for cell heights graph; N=3 for HBSS phagocytosis graph. n = 80 cells per condition for cell height (B) or n = 15 fields of view per condition per experiment for phagocytosis (C). Statistical analysis was performed utilizing the total sum n values per condition across all N. Statistical analysis was assessed using unpaired t tests: ****$p < 0.0001$. Error Bars represent SEM.

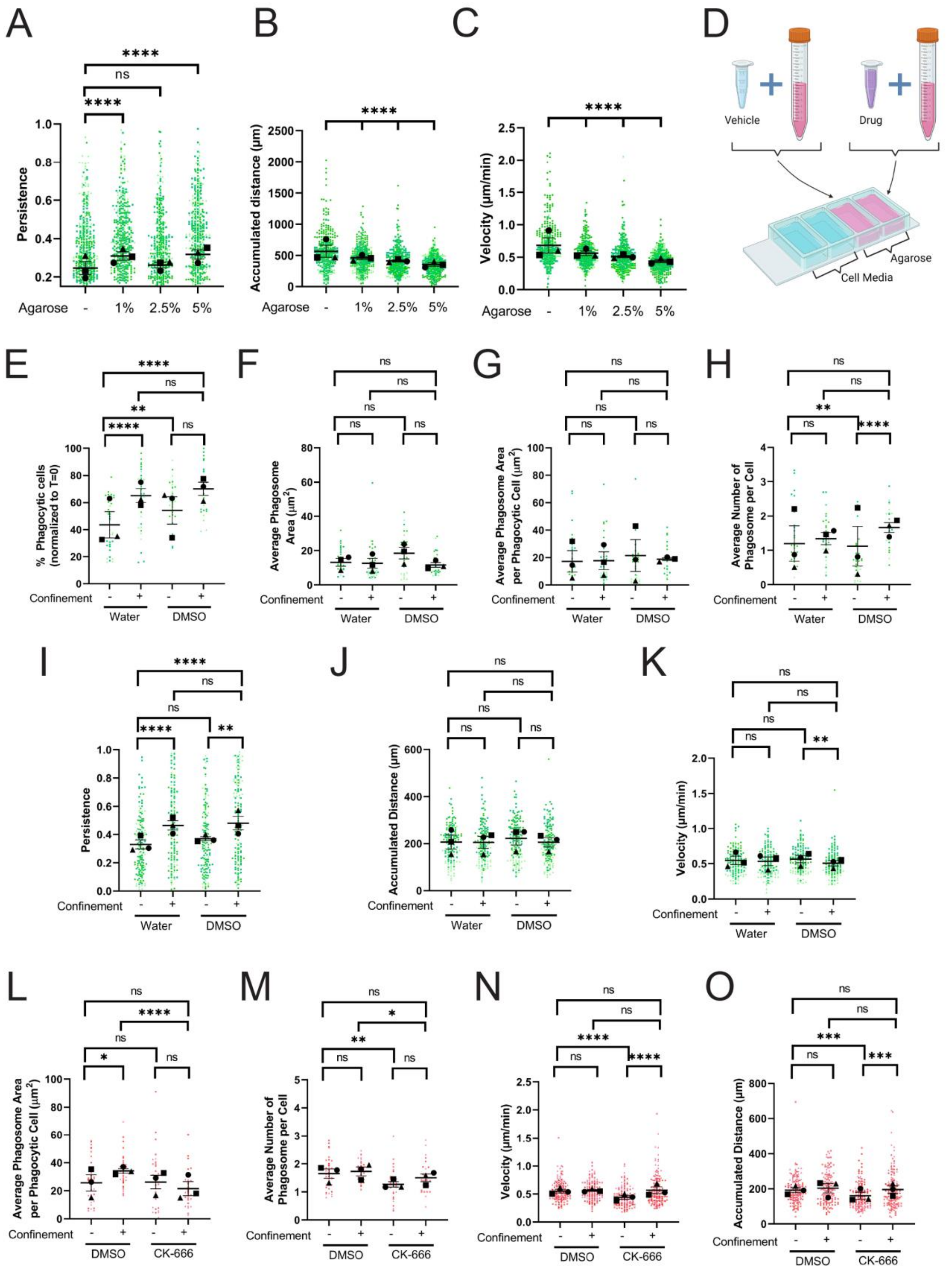

Biology Open • Supplementary information

Fig. S3. Controlling for agarose stiffness and DMSO influence on motility and phagocytosis. A) Persistence of the cells moving randomly (e.g. not post-phagocytic) in media, under 1% agarose, 2.5% agarose, or 5% agarose. B) Velocity (in µm/min) of cells moving in media, under 1% agarose, 2.5% agarose, or 5% agarose. C) Maximum total accumulated distance traveled (µm) for cells moving in media, under 1% agarose, 2.5% agarose, or 5% agarose. D) Schematic depicting how drugs were added into the agarose. Aliquots of 1% agarose were created and then vehicle or drug was added at the corresponding molarity before the agarose was poured into the confinement wells. E-K) These cells were either treated with water or DMSO. E) The percentage of fluorescent cells in a field of view, normalized to T=0. F) Average phagosome size (μm^2). G) Average phagosome area per phagocytic cell (μm^2). H) Average number of phagosomes per phagocytic cell. I) The persistence of the cell during the length of its track. J) Velocity of the cells migrating (µm/min). K) The maximum accumulated distance (µm) that the cell traveled during its tracking. L-O) These cells were either treated with DMSO or CK-666. These graphs relate to the experiments presented in Figure 3. L) Average phagosome area per phagocytic cell (μm^2). M) Average number of phagosomes per phagocytic cell. N) Velocity of the cells migrating (µm/min). O) The maximum accumulated distance (µm) that the cell traveled during its tracking. For all graphs, black points demonstrate experiment means, colored points demonstrate individual cell values for each run. N = 3 experiments for each graph; n = 15 fields of view for each condition per experiment for phagocytosis (E-H, L-M) or n = 80 cells for each condition per experiment for migration (A-C, I-K, N-O). Statistical analysis was performed utilizing the total sum n values per condition across all N. Statistical analysis was assessed using the Kruskal–Wallis with Dunn multiple comparisons test correction: ns = not significant, *$p < 0.05$, **$p < 0.01$, ***$p < 0.001$, ****$p < 0.0001$. Error Bars represent SEM. Created in BioRender by Paulson, S. (2025) https://BioRender.com/0u6ac7s

Biology Open • Supplementary information

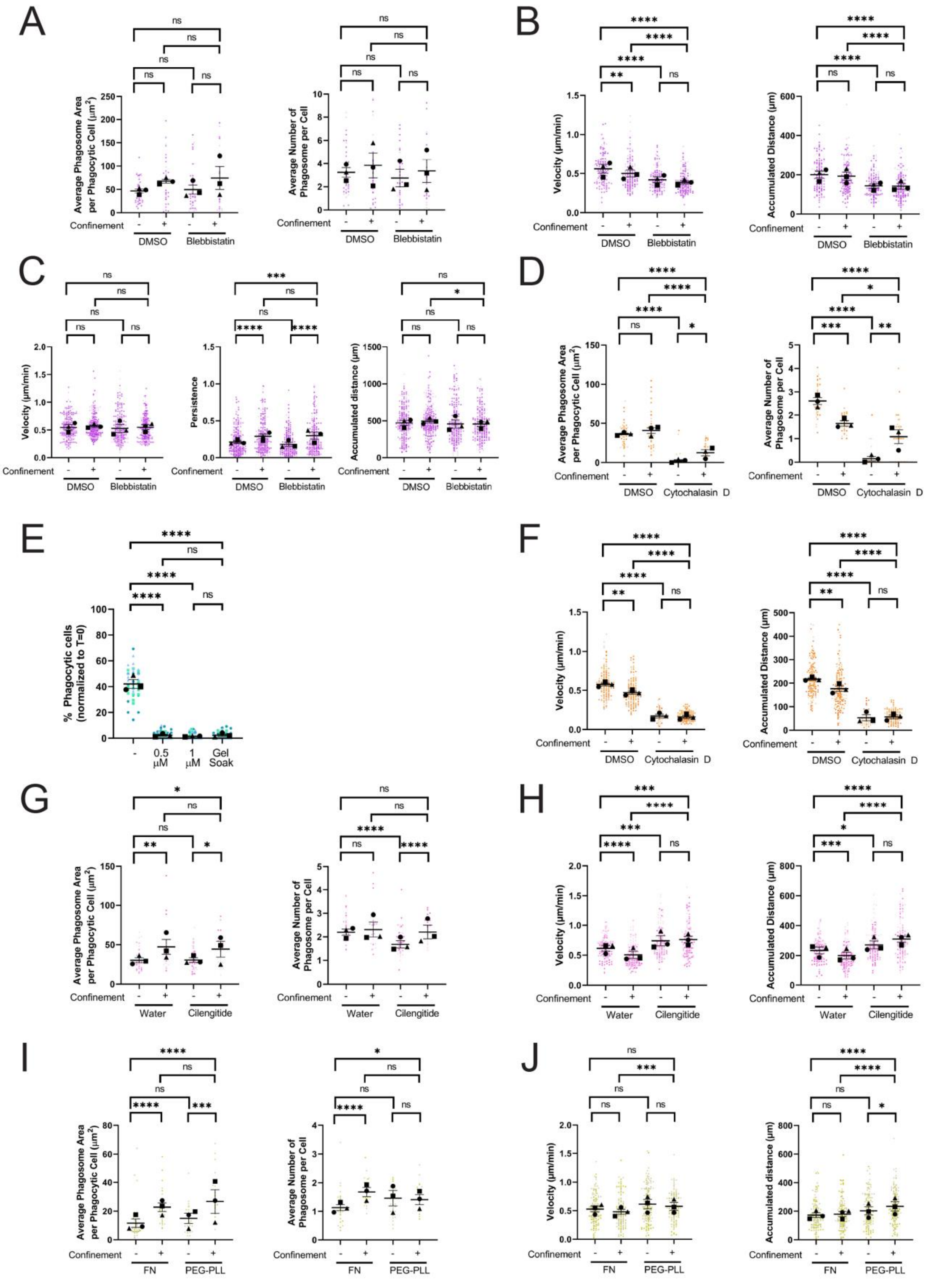

A
Average Phagosome Area per Phagocytic Cell (μm²)
Average Number of Phagosome per Cell
Confinement
DMSO
Blebbistatin
B
Velocity (μm/min)
Accumulated Distance (μm)
C
Velocity (μm/min)
Persistence
Accumulated distance (μm)
D
Average Phagosome Area per Phagocytic Cell (μm²)
Average Number of Phagosome per Cell
Cytochalasin D
E
% Phagocytic cells (normalized to T=0)
0.5 μM
1 μM
Gel Soak
F
Velocity (μm/min)
Accumulated Distance (μm)
G
Average Phagosome Area per Phagocytic Cell (μm²)
Average Number of Phagosome per Cell
Water
Cilengitide
H
Velocity (μm/min)
Accumulated Distance (μm)
I
Average Phagosome Area per Phagocytic Cell (μm²)
Average Number of Phagosome per Cell
FN
PEG-PLL
J
Velocity (μm/min)
Accumulated distance (μm)

Fig. S4. Additional phagocytic uptake and cell motility data related to Figures 4 and 5. A-B, D-F) These graphs relate to the experiments presented in Figure 4. G-J) These graphs relate to the experiments presented in Figure 5. A, D, G, I) Average phagosome area per phagocytic cell (left) and average phagosome number per phagocytic cell (right) when treated with the respective drug (Blebbistatin, Cytochalasin D, Cilengitide) or when plated on PEG-PLL. B, F, H, J) Velocity (µm/min) (left) and maximum accumulated distance traveled (µm) (right) of cells when treated with the respective drug (Blebbistatin, Cytochalasin D, Cilengitide) or when plated on PEG-PLL. C) Persistence (left), velocity (µm/min) (middle), and maximum total accumulated distance traveled (µm) (right) readings for cells with and without blebbistatin treatment when no beads are present. E) Percent phagocytosis measured at the 2-hour mark comparing Cytochalasin D gel-soaked media to cell media that has had DMSO or Cytochalasin D added directly to the well. For all graphs, black points demonstrate experiment means, colored points demonstrate individual cell values for each run. N = 3 experiments for each graph; n = 15 fields of view for each condition per experiment for phagosomes (A, D, G, I) and phagocytosis (E) or n = 50 cells for each condition per experiment for migration (B, F, H, J). Statistical analysis was performed utilizing the total sum n values per condition across all N. Statistical analysis was assessed using the Kruskal–Wallis with Dunn multiple comparisons test correction: ns = not significant, $*p < 0.05$, $**p < 0.01$, $***p < 0.001$, $****p < 0.0001$. Error Bars represent SEM.

Biology Open • Supplementary information

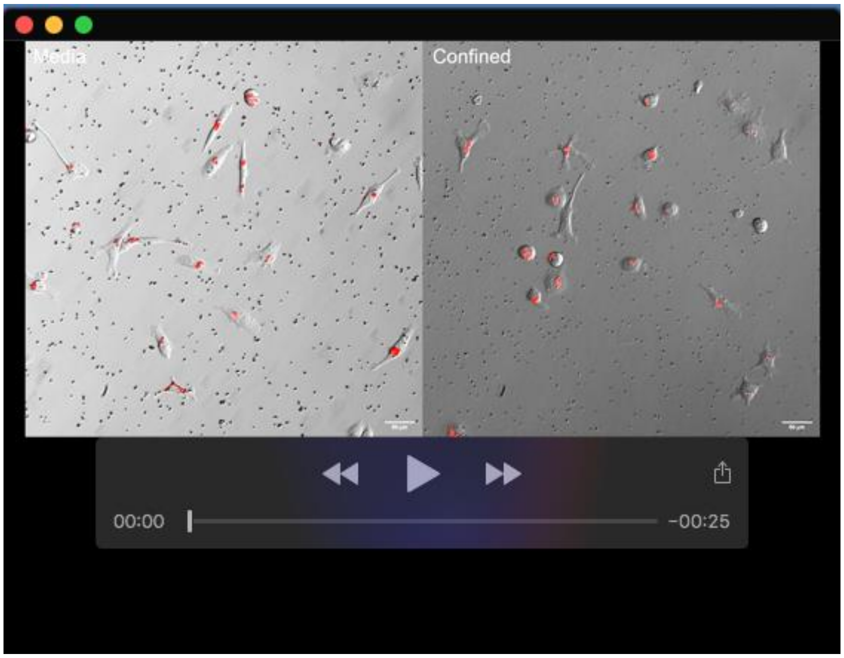

Movie 1. BV2 cells migrating in media vs confinement for 8 hours. Scale bar = 50 microns.

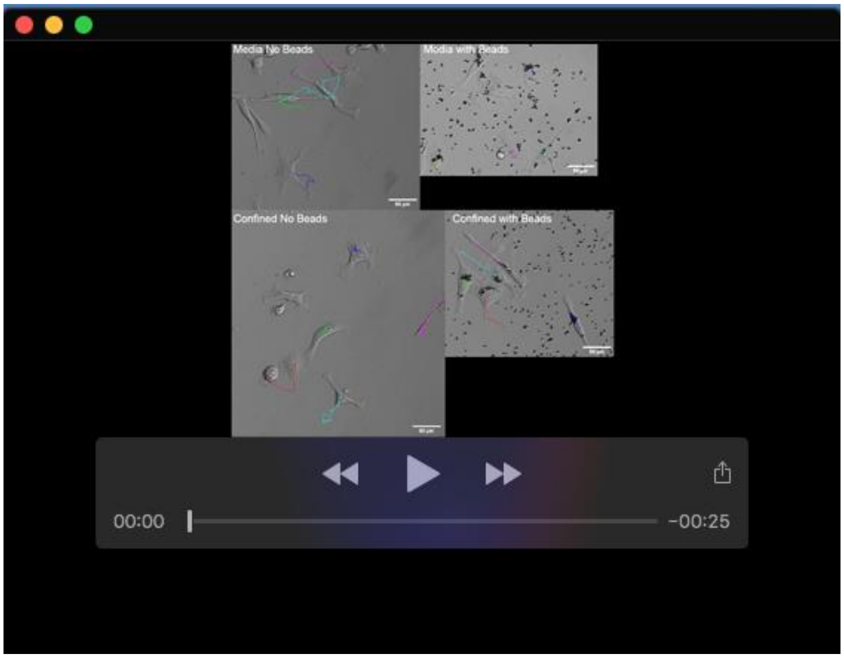

Movie 2. BV2 cells migrating in media vs confinement ± beads for 8 hours with manual cell tracking overlays. Tracking was stopped if cells began to divide. While these were used in our quantification, they do not persist to the end of each movie. These movies have been cropped to highlight individual cell trajectories. Scale bar = 50 microns.

Biology Open • Supplementary information

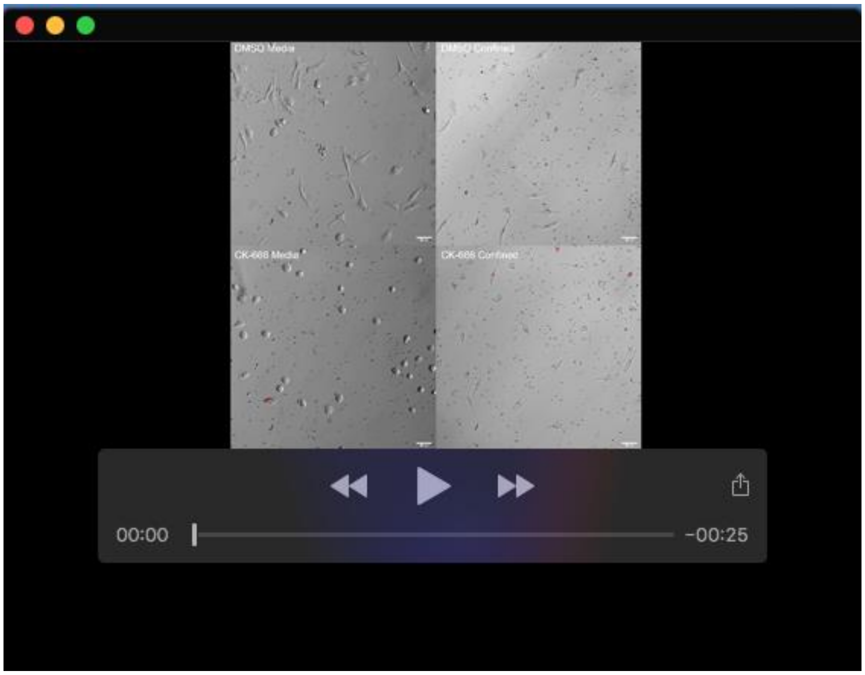

Movie 3. BV2 cells migrating in media vs confinement + CK-666 treatment for 8 hours. Scale bar = 50 microns.

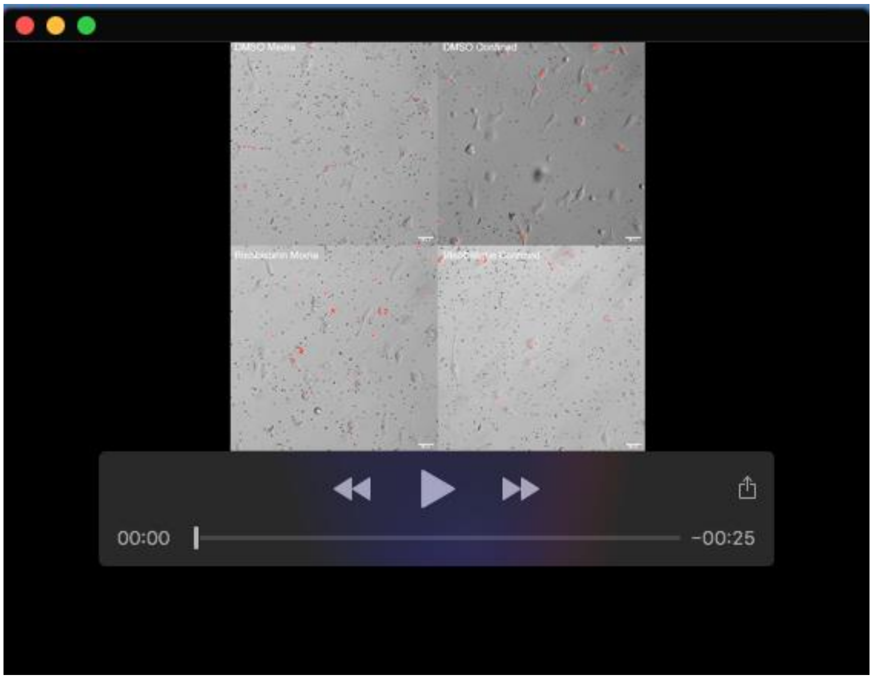

Movie 4. BV2 cells migrating in media vs confinement + Blebbistatin treatment for 8 hours. Scale bar = 50 microns.

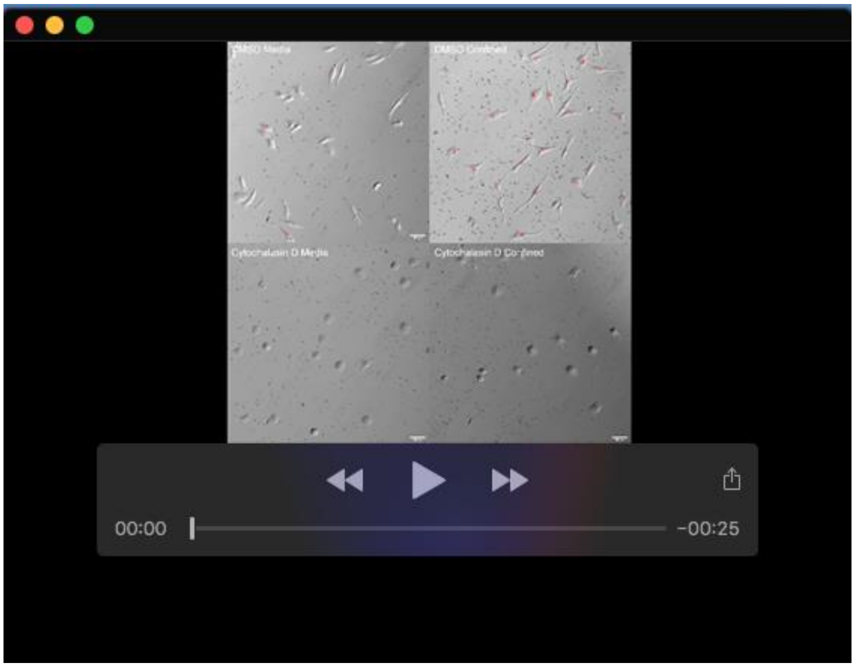

Movie 5. BV2 cells migrating in media vs confinement + Cytochalasin D treatment for 8 hours. Scale bar = 50 microns

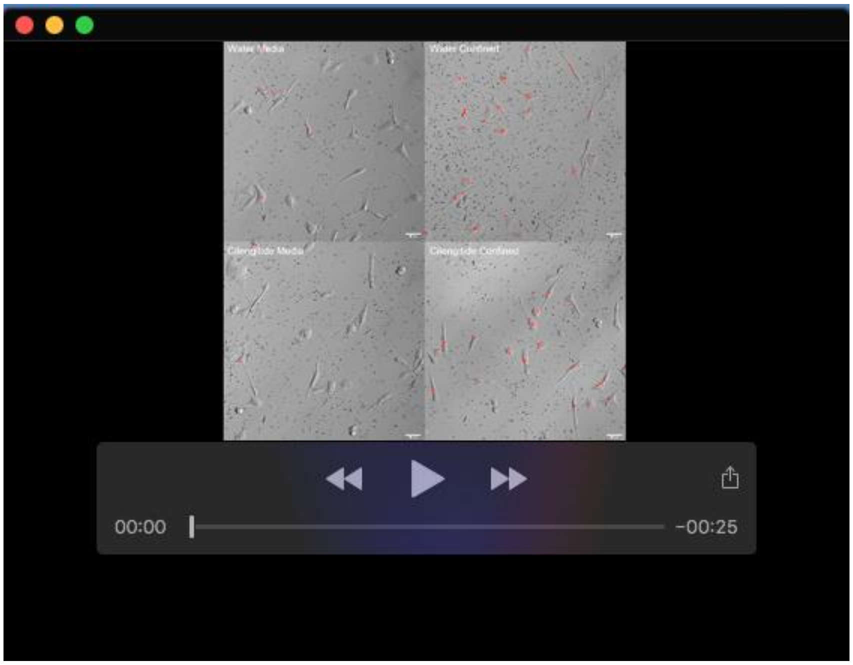

Movie 6. BV2 cells migrating in media vs confinement + Cilengitide treatment for 8 hours. Scale bar = 50 microns.

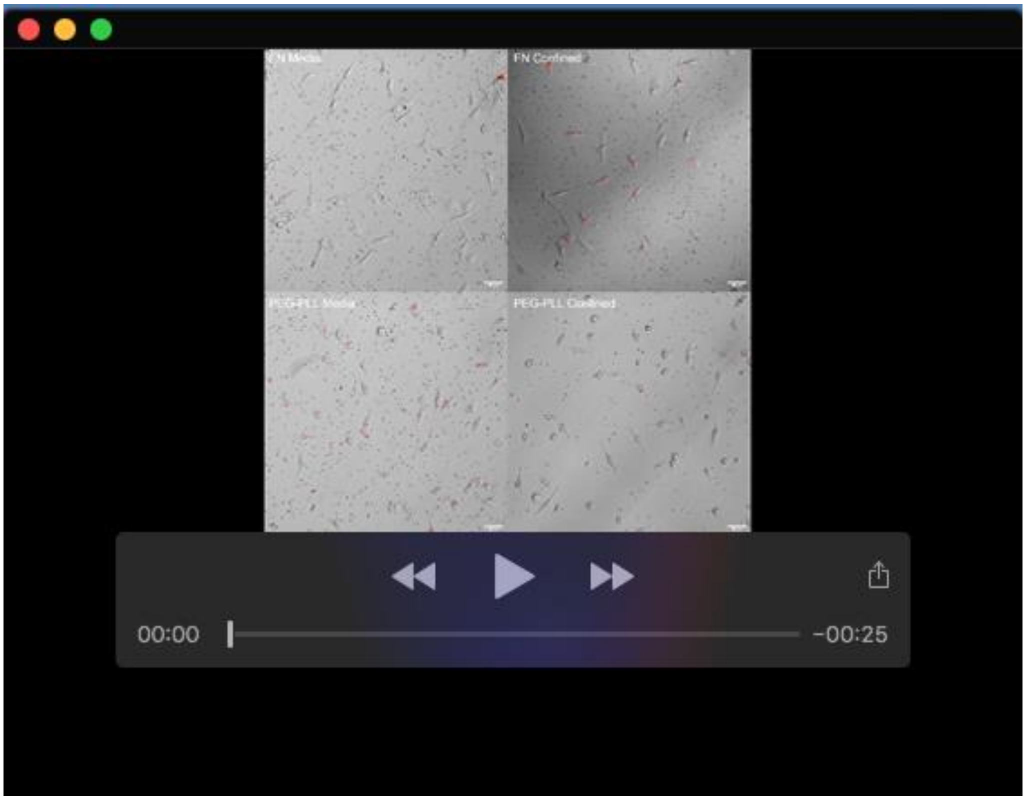

Movie 7. BV2 cells migrating in media vs confinement with FN or PEG-PLL coating for 8 hours. Scale bar = 50 microns.

Biology Open • Supplementary information
